# Small Animal Models of Respiratory Viral Infection Related to Asthma

**DOI:** 10.3390/v10120682

**Published:** 2018-12-01

**Authors:** Mingyuan Han, Charu Rajput, Tomoko Ishikawa, Caitlin R. Jarman, Julie Lee, Marc B. Hershenson

**Affiliations:** 1 Department of Pediatrics and Communicable Diseases, University of Michigan Medical School, Ann Arbor, MI 48109, USA; hanming@umich.edu (M.H.); crajput@umich.edu (C.R.); toishika@umich.edu (T.I.); cablake@umich.edu (C.R.J.); leejul@umich.edu (J.L.); 2 Department of Molecular and Integrative Physiology, University of Michigan Medical School, Ann Arbor, MI 48109, USA

**Keywords:** animal model, asthma, respiratory disease, rhinovirus, viral infection

## Abstract

Respiratory viral infections are strongly associated with asthma exacerbations. Rhinovirus is most frequently-detected pathogen; followed by respiratory syncytial virus; metapneumovirus; parainfluenza virus; enterovirus and coronavirus. In addition; viral infection; in combination with genetics; allergen exposure; microbiome and other pathogens; may play a role in asthma development. In particular; asthma development has been linked to wheezing-associated respiratory viral infections in early life. To understand underlying mechanisms of viral-induced airways disease; investigators have studied respiratory viral infections in small animals. This report reviews animal models of human respiratory viral infection employing mice; rats; guinea pigs; hamsters and ferrets. Investigators have modeled asthma exacerbations by infecting mice with allergic airways disease. Asthma development has been modeled by administration of virus to immature animals. Small animal models of respiratory viral infection will identify cell and molecular targets for the treatment of asthma.

## 1. Introduction

Asthma is a chronic inflammatory disorder of the conducting airways with reversible airflow obstruction and associated with mucus overproduction and airway remodeling. Though asthma is defined by typical symptoms such as cough, wheeze, chest tightness and/or shortness of breath, it has become increasingly recognized that asthma represents a heterogeneous disease with multiple phenotypes. Most children and roughly 50% of adult asthma patients have allergic asthma [1]. Allergic asthmatic patients have airway eosinophilic inflammation in parallel with increased type 2 T helper (Th2) cells and excessive production of type 2 cytokines such as IL-4, IL-5 and IL-13, as well as increased levels of IgE [2,3]. 

Respiratory viral infections are strongly associated with asthma exacerbations. Human rhinovirus (HRV) is most frequently-detected pathogen, followed by human respiratory syncytial virus (RSV), human metapneumovirus (hMPV), human parainfluenza virus (hPIV), human enterovirus (EV), human bocavirus (HBoV) and human coronavirus (HCoV) (Table 1)

Asthma development is most probably caused by an interaction of multiple factors, including, genetics, allergen exposure, microbiome and invading pathogens [32,33,34]. In particular, asthma development has been linked to wheezing-associated respiratory viral infections in early life [35,36,37]. It is unclear whether wheezing-associated respiratory viral infections cause asthma or are simply a marker of asthma susceptibility. 

Multiple infection models have been applied to explore the basic mechanisms of viral-induced asthma development and exacerbation. Primary epithelial cells, monocytes/macrophages and other cell types from either healthy control or asthmatic patients are usually collected and cultured for subsequent viral infection and measurement of cytokine/chemokine expression [38,39,40,41,42,43]. However, use of cultured cells limits the scope of research to cell-specific responses and prevents the study to cell-to-cell interactions or coordinated immune responses. Experimental infection of human subjects is available [44,45] but such studies may not always be feasible due to the safety concerns and difficulties in volunteer recruitment. Consequently, small animal models have been developed to better understand the mechanisms by which respiratory infection induce asthma and asthma exacerbation.

**Figure 1:**
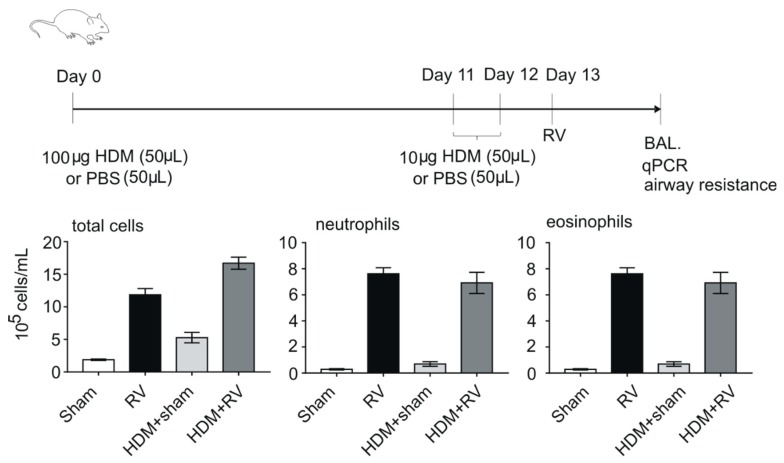
Example of an allergen sensitization and challenge model of allergic airways disease. Following the last challenge, mice are infected with human rhinovirus (HRV) to simulate an asthma exacerbation. The combination of house dust mite (HDM) exposure and viral infection induces an additive increase in airway eosinophils.

Asthma exacerbations are modeled in small animal models by sensitization and challenge with allergens such as ovalbumin (OVA) [4,46,47], dust house mite (HDM) [48,49,50] or cockroach allergen [51,52], thereby generating a mouse with allergic airways disease, followed by viral infection (Figure 1). Asthma development is modeled in small animals by administration of virus in immature mice [53,54,55].

To date, mice, rats, ferrets, hamsters and guinea pigs (Table 1, Table 2 and Table 3) have been extensively used for modeling human respiratory virus infections. The availability of immunological reagents and the convenience of genetic manipulation (transgenic and gene knockout mice) makes the mouse model the most commonly employed. Although most human respiratory viruses are not natural mouse pathogens and therefore require high-dose inoculums, mouse models have provided insights into mechanisms of pathology, immunology and vaccine biology. In this review, we summarize the small animal models of human respiratory viral infection relevant to asthma exacerbation and development, including HRV, RSV, EV-D68, hMPV and hPIV, as well as their contribution to the understanding of virus-induced asthma development and exacerbation. We also mention to mouse respiratory viruses which have been used to model viral-induced asthma, Sendai virus and pneumonia virus of mouse (PVM). 

Of note, we include only a limited discussion of HCoVs because most animal models have been established for the study of severe acute respiratory syndrome (SARS)-CoV and Middle East respiratory syndrome (MERS)-CoV which are less relevant to asthma. Finally, for the same reason, we do not include a description of animal models of influenza infection. 

## 2. Human Rhinovirus (HRV)

### 2.1. Virology and HRV-Induced Airways Disease

HRV is a small picornavirus grouped into the genus Enterovirus. HRV has an icosahedral, non-enveloped viral capsid carrying a positive sense, single-stranded RNA genome of approximately 7200 bp [78,79]. There are now 167 serotypes of HRV in three phylogenetic species, HRV-A, HRV-B and HRV-C [80]. HRV-A and HRV-B serotypes are also classified into two groups, major and minor, on the basis of receptor specificity, either intercellular adhesion molecule–1 (ICAM-1) [81,82,83] or low-density lipoprotein receptor (LDLR) [84], respectively. Cadherin-related family member 3 (CDHR3) serves as a receptor for the recently discovered HRV-C [85,86].

HRV, first identified in 1950s, is the most common cause of upper respiratory tract infection [87,88]. Accumulating evidence has determined the presence of HRV in the lower airway tract [89,90,91,92,93,94,95,96]. HRV is now recognized to be a common cause of asthma and COPD exacerbations. PCR-based studies examining the presence of viruses among patients when sick and healthy show a higher prevalence of viral infection during exacerbations. Outpatient children with asthma attacks show 62–81% positivity for viral infection versus only well children with 12–41% positivity [35,97]. Picornaviruses (primarily HRV) were detected in 65% of cases, coronaviruses in 17%, influenza and parainfluenza viruses in 9% and RSV in 5% [35]. Similar studies have been done in hospitalized children and adults [37,98,99,100,101,102,103,104]. Adults show a slightly lower number of viral infections during exacerbations. Finally, 22 to 64% of patients with COPD exacerbations are positive for virus versus 12 to 19% of non-exacerbating subjects [105,106,107,108,109]. In these studies, HRV is responsible for about half of the viral infections. The prevalence of rhinovirus may be even higher depending on the time of year. A recent study detected on HRV in 82% of all children admitted to an emergency room for acute asthma between January and July [97,110]. It should be noted that people with atopic asthma are not at greater risk of HRV infection than healthy individuals but suffer from more frequent, severe and longer-lasting lower respiratory tract symptoms [111].

In addition to its role in exacerbation of chronic airways diseases, accumulating evidence suggests a link between early-life wheezing-associated HRV infection and later asthma development. In a cohort study in Finland, HRV was detected in one third of infants hospitalized for wheezing and 60% of HRV-positive cases were diagnosed with asthma 6 years later [112]. According to the COAST (Childhood Origins of Asthma) birth cohort study, HRV wheezing illness during the first year of life were more associated with persistent wheezing at age 3 than HRSV and allergen sensitization [113]. Furthermore, in the same study, the following investigations showed nearly 90% of these patients with HRV-associated wheezing at age 3 had asthma developed at 6 years of age [114]. Finally, HRV was a significant risk factor for wheezing at age 13 whereas RSV was not [115].

### 2.2. Animal Models of HRV Infection

The development of a small animal model is useful to understand the pathogenesis of HRV infection. A major obstacle in developing a small animal model is that there are no known murine rhinoviruses. Due to poor sequence homology between human and mouse ICAM-1, major group serotypes like HRV-16 fail to infect mouse cell lines without artificial expression of human ICAM-1 or chimeric ICAM-1 with human domains [116,117]. On the contrary, the minor receptors, members of the LDLR superfamily, are evolutionarily highly conserved throughout species and a minor group serotype HRV-A1A infects mouse fibroblast cells without adaptation [118]. In addition, another minor group serotype HRV-A1B infects mouse cell lines and replicates more efficiently in mouse lower respiratory epithelial cell lines LA-4 and Mad-C3 than in the mouse fibroblast cell line (L) [117]. To improve replication of minor group HRVs in mouse cells, serial passages have been performed in mouse cell lines, alternately between mouse and human cell lines, or in vivo [119,120]. In addition, mouse L cells stably expressing human ICAM-1 (ICAM-L cells) have been used for HRV-A16 adaption [116]. The resulted mutations in the sequence encoding for 2C and 3A viral protein account for the adapted phenotype for HRV-A16 [116] and HRV-A1A [119], respectively. However, these adapted viruses did not show improved replication in vivo.

Based on the in vitro findings, experimental animal models using either minor-group HRV strains in wild-type mice or major-group HRVs in transgenic mice expressing human ICAM-1 have been developed [4]. High doses (5 × 10^6^ TCID50, tissue-culture infective dose) of HRV-A1B or HRV-A16 are needed to induce extensive peribronchial and perivascular inflammatory infiltrates, chemokines, pro-inflammatory cytokines and interferons (IFNs) [4,5]. The requirement for high titer HRV-A16 in transgenic mice expressing human ICAM-1 suggests there are other factors which limit replication in mice besides the viral receptor. HRV-A1B infection also increases the airway hyperresponsiveness to methacholine [5]. Although negative-stranded genomic RNA is detectable, indicative of virus replication, the mouse is only “semi-permissive” to HRV infection, based on the limited viral replication and rapid decline in viral RNA and titer within 24–48 h after infection [4,5,119]. The respiratory tract mucosal barrier may account for the declined viral titers, which may prevent the initial receptor binding of HRV or the later release of progeny from infected epithelial cells. Mice pretreated with hypochalorous acid maintain viral titers of a mouse-adapted HRV strain for 24 h and show a 1-log decrease after 48 h post infection in comparison with a 3-log decrease in non-pretreated mice [119]. Use of HRV animal models has elucidated important signaling pathways, proteins and receptors involved in HRV-mediated airway inflammation and airway hyperresponsiveness in naïve mice, including phosphatidylinositol 3-kinase [5], E3 ubiquitin ligase midline 1 (MID1) [49], tumor necrosis factor-related apoptosis-inducing ligand (TRAIL) [50], C-X-C motif chemokine receptor (CXCR) 2, tumor necrosis factor receptor (TNFR) 1 [121], melanoma differentiation-associated gene (MDA)-5, the adaptor protein for Toll-like receptor (TLR)-3 [122], chemokine (C-C motif) ligand (CCL)-7 [123], IFN regulatory factor (IRF)-7 [123] and TLR2 [124]. HRV-A1B infection of mice deficient in Tbet, a transcription factor required for Th1 differentiation, exhibits T helper cell-dependent airway eosinophilia and mucus production, suggesting that Th2 responses play an important role in driving features of allergic airway diseases during asthma exacerbations [125].

The cotton rat has recently been used to model HRV infection. In the cotton rats, major group strains HRV-A16 and HRV-B14 infect and replicate better than minor group HRV-A1B [6,7]. The receptor employed by these major group HRVs has not yet determined. Of note, currently, there is no available animal model for HRV C strains.

### 2.3. Models of HRV-Induced Asthma Exacerbation

**Figure 2:**
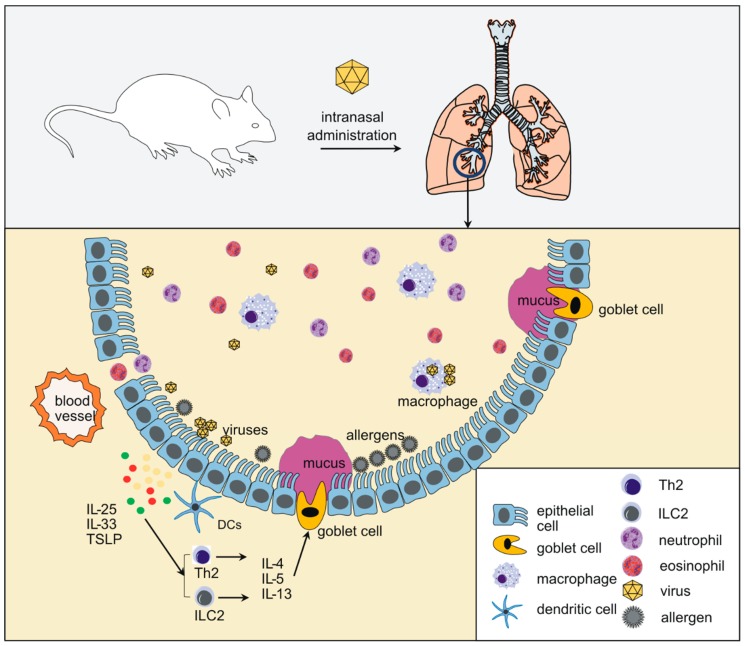
Array of airway cells involved in the response to viral infection. Viruses replicate in the airway epithelium and epithelial cells secrete chemokines which attract innate immune cells to the airway. The airway epithelium also includes resident dendritic cells that respond to allergens, pathogens and damage signals. Among the other innate immune cells shown to be activated after viral infection are exudative macrophages (also called inflammatory monocytes) and innate lymphoid cells. Epithelial-derived innate cytokines (IL-25, IL-33 and TSLP) play a special role as they may activate type 2 helper T (Th2) cells and type 2 innate lymphoid cells (ILC2s) which elaborate type 2 cytokines (IL-4, IL-5 and IL-13) leading to mucous metaplasia. Thus, under certain circumstances, initiation of type 2 inflammation by viruses may not require allergen exposure.

A major focus of animal models has been to elucidate the immune responses to HRV in mice with allergic airways disease. Allergen sensitization and challenge with multiple doses of either ovalbumin (OVA) or house dust mite (HDM) are performed before HRV infection [4,46,47]. Following OVA sensitization, HRV infection triggers eosinophilic inflammation and airway hyperresponsiveness, along with increased expression of mucins (Muc5AC and Muc5B), eotaxin-1/CCL11, IL-4 and IL-13 [4,46,47]. In this model, the major cellular source of CCL11 is surprisingly the activated CD11b+ exudative macrophage (Figure 2) rather than airway epithelial cells [47]. Further, the macrophage plays an indispensable role in eosinophilic infiltration and airway responsiveness in HRV-infected OVA-treated mice [46,47]. Administration of clodronate to deplete macrophages or neutralizing antibody against CCL11 each attenuate HRV-induced airway eosinophilia and responsiveness in OVA-sensitized mice [46,47]. CD11b+ cells from OVA-treated, HRV-infected CD11b-DTR mice show M2 polarization and depletion of CD11b+, IL-13-producing cells in, decreased airway inflammation and responsiveness [126]. HRV colocalizes with CD68+ CD11b+ macrophages following experimental infection in humans, confirming that macrophages play a role in human asthma exacerbations [127]. HRV infection has also been shown to abrogate inhaled OVA-induced tolerance by suppressing the generation of forkhead box protein 3 (Foxp3) + T regulatory (Treg) cells through IL-33, thymic stromal lymphopoietin (TSLP) and OX40 ligand (OX40L) signaling [128]. IL-33 and TSLP, along with IL-25, are epithelial-derived innate cytokines which stimulate production of type 2 cytokines via stimulation of Th2 helper cells and type 2 innate lymphoid cells (ILC2s, Figure 2). In addition, HRV infection of HDM-treated mice induces plasmacytoid dendritic cell (pDC) recruitment to the lung and pDC abrogates HRV-induced inflammation. IL-25 was also induced by allergen challenge and HRV infection and conditioned pDCs for pro-inflammatory function [129]. Sputum pDC numbers were also increased during human asthma exacerbations, confirming the value of the mouse model.

Aeroallergen exposure using HDM has been considered more clinically relevant to HRV infection and asthma exacerbation, as sensitization can be accomplished through the airways [130]. However, different HDM exposure methods may generate inconsistent outcomes. After ten consecutive days of intranasal exposure to 25 μg HDM, a classic Th2-driven response was not observed and HRV infection caused additive effects on airway neutrophilic inflammation as well as IgE level and airway responsiveness [130]. On the contrary, in mice receiving intranasal exposure of high-dose HDM at early time points and multiple treatments of low-dose HDM at later time points, HRV triggers both type 1 and type 2 responses including IL-4 and IL-13 expression, eosinophilic inflammation and airway hyperresponsiveness [48,49,50,131]. Using this HDM model, HRV infection triggers neutrophil extracellular traps (NETs)-related dsDNA release which is consistent with experimental human infection [48]. Finally, as an alternative method, dsRNA has been administered to HDM-treated mice to simulate viral-induced asthma exacerbation [128].

### 2.4. Models of HRV-Induced Asthma Development in Immature Mice

Genetic factors and early-life airway exposures to aeroallergens and respiratory viral infections predispose children to asthma development in later life. The immature immune system is qualitatively different from the adult, refractory to type 1 responses but permissive to type 2 responses [132]. Immature mice (younger than 7 days of age) have been used as an age-appropriate model to study virus-mediated chronic immunopathology and asthma development. A delayed and prolonged type 2 immune response which is featured with increased expression of IL-4, IL-5 and IL-13, as well as mucous metaplasia and airways hyperresponsiveness occurs in HRV-infected immature but not in adult mice [53,56]. On the contrary, type 1 response is attenuated in HRV-infected neonatal mice [56,132]. Thus, there is a developmental difference in the response to HRV. IL-13 has been shown to play a role in mucus metaplasia [133] and administration of anti-IL-13 neutralizing antibody can reduce the asthma-like phenotype following early-life HRV infection [53].

The cellular source of IL-13 in HRV-infected immature mice has been determined by flow-cytometry and type 2 innate lymphoid cells (ILC2s), not TCRβ+ cells, are the major cellular source of IL-13 secretion [56]. The requirement and sufficiency of IL-13-expressing ILC2s in the HRV-induced mucous metaplasia phenotype have been proven by administering g SR3335, a chemical inhibitor for ILC2-related transcription factor RAR-related orphan receptor alpha (RORα) and adoptive transfer of ILC2s to the lung immature and adult recipients, respectively [134]. SR3335 treatment decreases the number of lung ILC2s as well as mucous metaplasia [134]. Adoptive transfer of ILC2s causes mucous metaplasia in both immature and adult mice [134]. Increased expression level of the epithelial-derived innate cytokines IL-25, IL-33 and TSLP is observed in HRV-infected immature mice [55]. Treatment with anti-IL-25 or anti-IL-33 neutralizing antibody, or TSLP receptor (TSLPR) KO attenuates asthma-like phenotype and ILC2 expansion in HRV infected immature mice [55]. Finally, treatment of HRV-infected mice with recombinant IFN-γ, a classic type 1 cytokine, while not blocking expression of the epithelial-derived innate cytokines, inhibits ILC2 proliferation and activation as well as development of the asthma-like phenotype [132].

## 3. Enterovirus D68 (EV-D68)

### 3.1. Virology and EV-D68 Disease

Since the discovery in California in 1962 from hospitalized children [135], EV-D68 infection has been considered rare until a worldwide upsurge in detection of EV-D68 in the last decade [136,137,138,139,140]. The Morbidity and Mortality Weekly Report (MMWR) in 2011 highlighted EV-D68 as an increasingly recognized cause of respiratory illness, with six clusters of EV-D68 associated respiratory illness reported from Asia, Europe and the United States between 2008–2010 [141]. In 2014, the United States experienced a nationwide EV-D68 respiratory disease outbreak with over 1150 cases reported [142,143]. The epidemiologic study reveals EV-D68-associated respiratory illness was predominantly reported in children with a median age of 5 years, especially in the preschool and school aged children [142]. However, the age of those reported ranged widely from 3 days–92 years. Of note, more than half of children confirmed EV-D68 infection have a prior history of asthma [142,144]. Common symptoms include dyspnea, cough, wheezing and fever. In 2016, 138 cases of EV-D68 were reported to the National Respiratory and Enteric Virus Surveillance System (NREVSS) [145].

In addition to acute respiratory infection, cases of AFM were reported coincident with the 2014 EVD-68 outbreak [146,147]. The paralysis occurred primarily in children and many of them experienced respiratory illness before the onset of the limb weakness. A case-control study from the 2014 AFM cases in Colorado suggested an association of EV-D68 [148].

EV-D68 is a non-polio human enterovirus belonging to Group D, the enterovirus genus, whose members are characterized by a small icosahedral viral capsid and with a single positive-stranded RNA genome. EV-D68 strains consist of four major genetic clades A, B, C and recently suggested clade D, as determined by phylogenetic analysis of‘ the VP1 gene [136,140,149]. Clade B has three subclades (B1, B2 and B3). EV-D68 shares structural [150] and some biologic features [151] with HRVs. HRV-87 was subsequently identified as a EV strain by molecular diagnosis and antigenic characterization [152,153,154]. Sialic acid was initially considered important for the EV-D68 entry into the permissive cells with a preference of sialic acid with an α-2,6 linkages [155]. However, a later report suggests both α2,6- and α2,3-lined sialic acid function as cellular EV-D68 receptors and sialic acid-independent EV-D68 variants have been identified [156]. Furthermore, intercellular adhesion molecule 5 (ICAM-5) has recently been identified to be indispensable for the entry of both sialic acid-dependent and independent EV-D68 and soluble ICAM-5 fragment inhibits EV-D68 infection in primary human bronchial epithelial cells, primary rat neurons and mouse brain tissue [8].

### 3.2. Animal Models of EV-D68

The prototype of EV-D68, the Fermon strain, was isolated from the respiratory specimen of pediatric patients with lower respiratory tract illness, indicating its tropism targets the respiratory tract [135]. The ability of EV-D68 to infect the mouse respiratory tract was initially evaluated using cotton rats [11]. Three EV-D68 isolates, the prototype Fermon strain, pre-outbreak isolate VANBT/1 and outbreak isolate US/MO/14-18949, were given through intranasal route with a titer of 106 TCID50. These three isolates showed varied replication ability, with the VANBT/1 isolate showing the most potent replication in both nose and lung. Resembling the mouse model of HRV infection, however, EV-D68 replicated to a limited extent in the rat airway and the infectious virus and amount of negative strand genomic RNA were almost undetectable at 48 h post-infection. Nasal inoculation of cotton rats with VANBT/1 showed significant induction of pulmonary cytokine mRNA expression (CCL2, CCL5, CXCL1, CXCL10, IL-6, IFN-β and IFN-γ) and histologic evidence of peribronchiolitis and alveolitis [11]. In another study using adult BALB/c mice, EV-D68 (US/MO/14-18947 strain) viral RNA was detected in lungs up to 4 days after infection with a gradual decline starting from 12 h post-infection. Compared with HRV-A1B infection, EV-D68 induced higher IL-17 mRNA and protein, neutrophilic inflammation and airway responsiveness, due to an increased number of IL-17-producing ILC3s and γδ T cells [9]. Higher nasal IL-17 mRNA levels were confirmed in human patients.

Cotton rat and mouse models for EV-D68 airway infection are asymptomatic. On the other hand, ferrets infected with EV-D68 intranasally show clinical signs of respiratory illness including cough, nasal discharge and dry nose [12]. A large amount of viral RNA was found in the feces, nasal swab, lymph node and lung, while the viral load was low in trachea, bronchoalveolar lavage fluid and blood. No virus was detected from the central nervous system or throat swabs. Interestingly, α2,6- but not α2,3-linked sialic acid showed intense co-localization with EV-D68 in the ferret lung [12]. Nasal infection of ferrets increased lung protein abundance of IL-1α, IL-5, IL-8, IL-12, IL-13 and IL-17a as well as alveolar inflammation and hemorrhage [12].

#### Enterovirus-D68 Acute Flaccid Myelitis (AFM) Model

An immature mouse model has been established to study the correlation between EV-D68 infection and AFM [10]. Five 2014 circulating strains and two prototype strains were used to intracerebrally infect 2 day old mice and four out of five of the 2014 circulating strains led to paralytic disease and/or neonatal death with varied disease rate, time course and mortality. After intracerebral injection of the MO/14-18947 strain, viral titers in the spinal cord peaked at day 4 and steadily dropped until day 12 post-infection. The onset of paralysis occurred 3–5 days after infection which was coincident with peak viral titer in the spinal cord. The paralysis produced in forelimbs and/or hindlimbs, with the former being the most commonly affected [10]. Alternative routes of EV-D68 infection, including intramuscular, intranasal and intraperitoneal infection, also caused paralysis but it was rare and delayed [10]. In a recent study, intraperitoneal infection of one-day-old Institute of Cancer Research (ICR) mice with EV-D68 strains US/MO/14-18947 and US/KY/14-18953 but not Fermon strain, caused limb paralysis and death which was age- and virus dose-dependent [157]. In addition, AG149 interferon receptor deficient mice also showed neurological and muscle disease after intraperitoneal infection of EV-D68 [158].

### 3.3. Animal Models of EV-D68 Induced Asthma Exacerbation

EV-D68 preferentially causes severe respiratory symptoms in children and adults that have a prior history of asthma [142]. Thus, in addition to naïve mice, HDM-sensitized and -challenged mice also been studied [9]. In mice with allergic airways disease, EV-D68 enhances allergen-induced type 2 inflammation with increased expression of lung IL-5, IL-13 and Muc5ac and augmentation of bronchoalveolar lavage fluid eosinophils and airway responsiveness [9].

## 4. Human Respiratory Syncytial Virus (RSV)

### 4.1. Virology and RSV Disease

Human respiratory syncytial virus (RSV) is the major cause of serious respiratory disease in infants and young children, usually manifested as a bronchiolitis with wheezing [159,160]. RSV also produces significant morbidity and mortality in elderly and immune compromised adults. Most infants are infected by 2 years of age, with the incidence of severe disease peaking between 6 weeks and 6 months [161,162]. RSV regularly re-infects older children and adults, causing colds and, in patients with chronic lung disease, exacerbations of asthma or COPD. As noted above, infants experiencing community RSV infection suffer from asthma-type symptoms like cough and wheeze which resolve by 13 years of age [115,163]. However, infants with severe RSV bronchiolitis requiring hospitalization may have an increased frequency of asthma in later childhood [164,165].

Human RSV is a member of the Pneumoviridae family, Orthopneumovirus genus, along with closely related Orthopneumoviruses, including bovine RSV, ovine RSV and pneumonia virus of mice (PVM) [166]. Orthopneumoviruses are enveloped viruses with the genome organized with a negative-sense, non-segmented RNA, which is about 15,000 nucleotides in length and encodes for 11 viral proteins. A two-step process is used for RSV entry, a viral glycoprotein-mediated attachment step and a fusion step through binding of the viral fusion protein (F protein) to the receptor nucleolin [167]. In the lower airway, the airway epithelium is the primary infection site and macrophages in the lung may be infected as well [168].

### 4.2. Experimental Animal Models of RSV

RSV was first isolated from a chimpanzee [169] and experimental infection of chimpanzees causes upper respiratory tract disease resembling RSV disease in humans [170]. However, evidence of lower airways disease was not detected. The non-human primate model has been extended to Cebus monkeys [171] and owl monkeys [172] and these models have been mainly used to evaluate vaccine efficacy and study vaccine-related pulmonary pathology (see review [173]). Starting from late 1970’s, small animal models of human RSV infection were developed using ferrets [17], cotton rats [14], inbred mice [13] and guinea pigs [15,174] (Table 1, Table 2 and Table 3).

In guinea pigs, human RSV infection causes acute bronchiolitis without clinical signs and weight loss [15]. Viral antigens appear primarily in the airway epithelium and alveolar macrophage. Interestingly, RSV persistence has been observed in infected lung of guinea pigs up to 60 days [73,174]. RSV infection also increases cytokine expression, lymphocytes, neutrophils, eosinophils and airway hyperresponsiveness occurs in naïve or OVA-sensitized guinea pigs [175,176]. In ferrets, human RSV infects both the upper and lower respiratory tracts and triggers production of IL-1α, IL-1β, TNF-α, IFN-γ, IL-17 and various chemokines [17,177]. Infectious RSV is cleared 9 days after infection [177]. Like guinea pigs, no clinical signs are observed in RSV-infected ferrets [17]. In lungs of RSV-infected ferrets, virus replication is age-dependent with highest titers in the youngest animal [17]. The cotton rat is also permissive for human RSV with a similar viral growth kinetic as the mouse, in which the viral titer peaks at day 4 and declines to undetectable at day 7 [14]. Unlike the ferret, RSV replication in the cotton rat is not age-dependent. Viral antigen primarily appears in the bronchial and bronchiolar epithelium but not trachea and alveolar cells [14]. The cotton rat is a standard model for evaluation of vaccines, antivirals and neutralizing antibodies [178,179,180].

Non-human orthopneumoviruses/natural host pairs, bovine RSV/calf and PMV/mouse, represent alternative approaches to the study of orthopneumoviruses infections. Besides fully permissive replication in their natural host, infection of bRSV and PMV induce a similar immune cell responses as human RSV infection, such as CD8 +T cells and pro-inflammatory cytokine and chemokine responses [181,182]. The cognate virus models of human RSV have been reviewed extensively by Taylor [173], Sacco et al. [183] and Bem et al. [184].

### 4.3. Human RSV Mouse Model

Compared to other small animal models, the human RSV mouse model has been widely used for the study of pathogenesis as well as vaccine development. This is based on the large pool of available immunological reagents and commercially available transgenic and gene-deleted mice. Long [13] and A2 strains [58] of RSV have been the standard laboratory strains employed to infect mice. In the lung, RSV titers peak on days 4 to 6 post-infection and decline to undetectable at day 11 [58,59]. Infectious virus is recovered from both upper and lower airways [58,60]. To visualize RSV in living mice, recombinant human RSV expressing the firefly luciferase has been constructed using the Long [185] and rA2-line 19F as backbones [186]. Bioluminescence signal has been detected in the nasal cavity and lungs of infected-mice [185,186]. In the lower airway, fluorescence staining reveals the presence of viral antigen in the bronchiolar epithelium and alveoli [58,59,65]. Perivascular and peribronchial lymphocytes and macrophage infiltrates progressively develop during RSV infection, which is age- and inoculum size-dependent [59,60]. Histological lung lesions has been noticed as well [58]. RSV infection can cause visible illness manifested by ruffled fur, reduced activity and weight loss in mice [60].

BALB/c is the most common mouse strain used in experimental infections of human RSV (See Table 1). The susceptibility of mice to human RSV is host-strain dependent. Twenty different strains of inbred mice have been screened initially by giving RSV Long strain to 3-day old mice intranasally [13]. Strain-specific differences in RSV susceptibility of immature mice have been observed with the sDBA/2N strain being most susceptible and two common strains, BALB/c and C57BL/6, having intermediate susceptibility [13]. In adult mice, AKR/J was the most permissive strain among eight examined eight strains, while the C57BL/6 is the most resistant [57]. F1 progeny from AKR/J and C57BL/6 mice inherit RSV resistance from the resistant C57BL/6 parent [57]. A genome-wide association study of disease following human RSV infection in 30 inbred strains of mice identified several potential genetic determinants for RSV susceptibility, including the macrophage receptor with collagenous structure (Marco), an innate immunity scavenger receptor [187]. In addition to genetic determinants, the age dependence of viral replication in the lung has been documented [58,60]. RSV replication in the lungs of older mice was significantly greater than younger mice using A2 strain infection [58,60,188], though RSV infection in newborn mice follows the same viral kinetics as in adults with peak viral load at four days post-infection [13,54,57,61,189,190]. Sex does not seem to change the susceptibility to RSV infection or viral replication [58].

Mouse models implicate specific subsets of pulmonary immune cells in the development of human RSV disease. Acute RSV infection causes pulmonary cytotoxic responses in the lung with increased populations of natural killer (NK) cells and CD8+ T cells [59,191,192]. CD8+ T and NK cells are involved in pathogen clearance and immunopathology during RSV infection [191,192]. RSV-specific memory CD8+ T cells cause lethal immunopathology upon RSV reinfection [193]. In contrast, RSV-specific CD60+/CD103+ tissue resident memory T cells confer protection against severe respiratory viral disease during RSV reinfection [194,195,196]. Regulatory T (Treg) cells also reduce the immunopathology and airway inflammation by limiting both innate and adaptive immunity such as suppression of antigen-specific CD8+ T cell responses and maintain Th1/Th2 balance [197,198,199,200]. Finally, conventional CD103+ DCs are essential to promote CD8+ T cell responses [201], whereas the plasmacytoid DC and its derived semaphorin 4a are indispensable for Treg expansion during HRSV infection [202].

Human RSV infection has been associated with increased IL-4 expression, suggestive of a type 2 inflammatory response [203,204]. However, experimental infection using the human RSV A2 strain does not trigger eosinophilic inflammation or abundant Th2 cytokine expression in adult BALB/c mice, which may be partially due to the function of Treg cells [200] and CD8+ T cells [59,62,205]. Priming the immune response by immunization with a formalin-inactivated human RSV vaccine or sensitization with the RSV attachment glycoprotein (G) augments the Th2 response and promotes pulmonary eosinophilia during RSV infection [63,64]. In these primed adult mice, CD8+ T cells downregulate Th2 pathology by suppressing Th2 cytokine expression and eosinophilic inflammation [59,62,205]. IFN-γ-expressing NK cells precede CD8+ T cells and are essential to recruit CD8+ T cells in the lung [23]. IL-12 treatment further augments NK cell IFN-γ expression which in return inhibits lung eosinophilic inflammation [206].

Different human RSV strains cause different phenotypic responses in adult mice. The Line 19 strain, isolated from the infant patient at the University of Michigan [207], induces significant dose-responsive airway hyperresponsiveness, mucus metaplasia and IL-13 expression in 6-week old BALB/c mice, whereas the A2 and Long strains do not [208,209,210]. Meanwhile, the expression of the innate cytokines IL-25 and TSLP are increased in Line 19 infected BALB/c mice [211,212]. Anti-IL-25 treatment or TLSPR KO blocks RSV Line 19-induced airway hyperresponsiveness and mucus production [211,212]. No significant viral replication difference between strains A2 and Line 19 has been observed. The fusion (F) gene of Line 19 strain has been identified as a mucogenic virulence factor and a recombinant virus rA2-Line 19F, carrying fusion (F) gene from the Line 19 strain induces airway hyperresponsiveness, mucus metaplasia and IL-13 expression as Line 19 does [209]. Strain-specific pathogenesis has been further confirmed using six different clinical isolates of the RSV antigenic subgroup A [65]. Infection of mature BALB/c mice with the A2001/2-20 isolate causes more weight loss, dyspnea, IL-13 and Gob5 expression, epithelial desquamation, bronchiolitis, mucus metaplasia and airway hyperresponsiveness than the other strains, while showing fewer IFN-γ expressing CD8+ T cells, which function to suppress RSV-induced Th2 responses [65]. Interestingly, A2001/2-20 showed several unique features the first day of infection, including significantly higher viral load, the presence of RSV antigen in the bronchiolar epithelium and perivascular edema [65]. Blocking IL-25 signaling attenuates allergic airway inflammation in RSV A2001/2-20-infected mice [211]. As was shown earlier in HRV-infected 6 day-old mice [55,56]. In addition, ILC2s have been determined to be the major early source of IL-13 production in A2001/2-20 infected adult mice, a process which is TSLP and IL-33 dependent [66]. The transcription factor signal transducer and activator of transcription 1 (STAT1) governs ILC2 expansion during A2001/2-20 infection [213].

### 4.4. Models of RSV-Induced Asthma Exacerbation in Mice with Allergic Airways Disease

Allergen sensitization has also been used to study the effects of human RSV on allergic responses and asthma exacerbation [214]. In OVA-sensitized adult mice, RSV infection prolongs airway lymphocytic inflammation, mucus deposition and methacholine-induced airway hyperresponsiveness (but not the number of airway eosinophils) compared to mock infection [214]. Expression of mucus-related genes Muc5ac and Gob-5 is also increased in OVA-treated, RSV-infected mice [215]. Interestingly, OVA-sensitized, RSV-infected mice also show increased expression of IL-17A, which is protective against Th2 allergic responses. Accordingly, IL-17A knockout mice show more IL-13 production and eosinophil infiltrates [216]. Paradoxically, infection with human RSV decreases house dust mite-induced eosinophils [217]. 

RSV-induced exacerbation of allergic airways disease has also been studied in mice exposed to cockroach allergen [51,52,218]. In these studies, RSV potentiates cockroach-induced type 2 inflammation. During RSV exacerbation, in vivo neutralization of a specific Notch ligand, Delta-like ligand-4, significantly decreased airway hyperresponsiveness, mucus production and Th2 cytokines [51].

### 4.5. Models of Human RSV-Induced Asthma Development in Immature Mice

Since human RSV infection in early life is suspected to play a role in the development of post-bronchiolitis wheezing and asthma, immature mouse models with mice age ranging from 1 day to three weeks have been used to closely mimic the interaction between RSV and the human infant immune system (see Table 1). During experimental infection, antigen-specific and IFN-γ expressing CD4+ and CD8+ T cells are recruited to the lung tissue and airway which peak 8–10 days post-infection [219,220]. However, T cell recruitment in the airway is reduced in RSV-infected immature mice compared to adults [220]. The CD8+ T cell response is also age-dependent and limited in 7-day old mice compared to mice older than 4 weeks [61,188]. Aged mice show a significant reduction in CD8+ T cells in response to RSV infection but cytokine production is preserved [221]. In addition, the epitope hierarchy of neonates is distinct from adults in human RSV-infected mice, with a codominant response against both KdM282-90 and DbM187-195 epitopes in neonates following HRSV infection, compared to an immunodominant response to the KdM282-90 epitope in adult [188]. In immature mice, the CD8+ T cell immunodominance hierarchy has been linked to the role of plasmacytoid DCs [222], which are quantitatively and functionally deficient compared to adults [201]. CD103low DCs, which represent the major fraction of CD103+ plasmacytoid DCs, are phenotypically immature with lower expression of lineage-defining and maturation markers and induce weaker CD8+ T cell specific responses [223].

Aside different cellular responses, a type 2 skewed immune response has been observed in human RSV-infected neonatal mice. Immature mice have shown reduced and delayed IFN-γ responses following RSV infection [61,224]. By contrast, compared to 3-weeks old mice, mice less than 1-week old showed more IL-13 expression along with increased number of mucus-producing cells and airway tissue eosinophilia during RSV infection [224]. The recombinant HRSV rA2-Line 19F has been used to optimize the neonatal RSV infection model [54]. Similar to HRV neonatal infection [56], ILC2s are increased following neonatal HRSV infection but not in adult mice, which is in agreement with an age-dependent epithelial-derived IL-33 expression [225].

The immature infection model has also been extended to study recurrent RSV infection. Mouse studies show that the age of initial infection is a risk factor for subsequent RSV-mediated disease. Seven-day old mice infected with human RSV show more severe weight loss, airway hyperresponsiveness and airway inflammation (including neutrophils, eosinophils, CD8+ T cells and type 2 cytokine expression) during RSV re-challenge in adulthood compared with mice undergoing primary infection at four weeks [61,224,226]. Major histocompatibility complex (MHC) haplotype, CD4+ T cells, CD8+ T cells, NK cells, macrophages, type 1 IFNs and pDCs all play important roles in the response to reinfection [226,227,228,229,230]. STAT6 inhibitory peptide treatment of young mice at the time of primary RSV infection attenuates the response to RSV reinfection [231].

As noted above, infants experiencing community RSV infection suffer from asthma-type symptoms like cough and wheeze which resolve by 13 years of age [115,163]. However, a cohort of Swedish infants with severe RSV bronchiolitis requiring hospitalization later developed asthma which continued into young adulthood [164,165]. Young adults also showed increased prevalence of clinical allergy, suggesting that early-life RSV infection could alter responses to allergen exposure. Consistent with this, early-life human RSV infection predisposes mice to enhanced allergic airway disease and Th2 responses to allergen exposure in adulthood [232]. To the contrary, primary RSV infection protects adult mice from OVA-induced allergic responses by inhibiting ILC2 [233]. Moreover, repeating RSV infection in OVA-tolerized infant mice impairs Treg function by inducing a Th2-like effector phenotype, resulting in allergic airway disease in response to later OVA exposure [234].

### 4.6. PVM Model of Asthma Development

Like human RSV, PVM is a member of the Pneumoviridae family, Orthopneumovirus genus. Infection of mice with PVM therefore represents a useful model of human RSV infection [18]. Similar to HRV infection [129], there is a marked infiltration of pDC into the airways following PVM infection which is attenuated in TLR7- and MyD88-deficient mice [235]. In another study, cockroach extract-induced IL-33 expression dampened antiviral immunity to subsequent PVM infection [236], providing a mechanism which could predispose asthma patients to more symptomatic viral infections.

## 5. Human Metapneumovirus (hMPV)

### 5.1. hMPV Virology and Disease

First isolated from patients with lower respiratory tract infections in the Netherlands in 2001, hMPV was retrospectively identified in samples collected in the 1950’s [237]. Closely related to RSV, hMPV is a member of the Pneumoviridae family, Metapneumovirus Genus. hMPV also causes respiratory infections in human of all ages, primarily in young children and in immunocompromised individuals, with clinical signs of pneumonitis, bronchiolitis and acute wheezing [238,239,240,241]. In addition, hMPV infection has rarely been associated with asthma exacerbation in both children and adult [240,242,243]. hMPV contains a non-segmented, negative-sense RNA genome approximately 13500 nucleotides in length [244]. The genomic organization for hMPV is similar but not identical to that of RSV. hMPV lacks the non-structural proteins NS1 and NS2 of RSV and the gene order of RSV and hMPV varies significantly [244].

### 5.2. Animal Models of hMPV Infection

Non-human primates and small animal models for hMPV were established immediately after the first identification of hMPV [22,245]. During intranasal infection, hamsters and ferrets are most permissive tor hMPV replication [21,22]. BALB/c mice and the cotton rat have intermediate susceptibility for hMPV infection [21,22]. In the cotton rat, hMPV titer peaks around 4 days after infection and viral antigens are detected exclusively in respiratory epithelial cells [20,21]. hMPV subtype A strain grows better than the subtype B strain in cotton rats [20].

In BALB/c mice, hMPV replication shows a biphasic growth kinetic with two peaks of viral titer at 7 days and 14–28 days after infection [19,67]. Similar to RSV, hMPV can persist in the lung with recovered infectious virus up to 60 days and detectable viral genomic RNA over 180 days [19]. The lung persistence of HMPV has been associated with the HMPV infection of PGP9.5+ neuronal cells [67]. Around the peak time of hMPV replication, infected-mice show clinical symptoms consisting of breathing difficulties, ruffled fur and weight loss [68]. In the airway, hMPV causes increased mucus production, airway hyperresponsiveness and airway obstruction after the primary infection [19,69,246,247]. Neutrophil and mononuclear cell infiltration [19,68] as well as increased CD8+ but not CD4+ T cells have been seen in hMPV infected mice [248]. Neutrophil depletion using antibody against Ly6Ghigh neutrophils leads to increased pulmonary inflammation and more severe clinical disease during hMPV infection, even though neutrophils are not associated with hMPV clearance [249]. Depletion was associated with an increase in γδ T cells and deficiency of these cells reduced lung pathology, suggesting they play a deleterious role.

T cell depletion assays suggest that CD4+ and CD8+ T cells cooperate synergistically in hMPV clearance but CD4+ rather than CD8+ T cells efficiently enhance clinical disease and lung pathology during primary infection [250]. hMPV infection increases both Th1 (IFN-γ) and Th2 cytokines, including IL-4 and IL-5 [246,247,248]. Aging is another important factor affecting hMPV pathogenesis. The aged mice showed higher clinical severity which is coincident with attenuated production of virus-specific antibody and IFN-γ and increased IL-4 expression [251].

Since hMPV is closely related to human RSV, the pathology and immune responses to the hMPV D03-574 and RSV A2 strains have been directly compared in BALB/c adult mice [252]. hMPV infection causes more severe airway obstruction, weight loss and histopathology. In addition, more neutrophils and activated NK cells were recruited in hMPV-infected mice, which correlated with increased IL-6, TNF-α and CCL2/MCP-1 [252].

Though hMPV infection has been linked to asthma exacerbation, few studies have been performed to elucidate underlying mechanisms. hMPV infection causes eosinophilic and increased Th2 cytokines in mice immunized with the inactivated hMPV [253]. TSLP expression is increased in hMPV infected mice, while TSLPR-/- mice showed reduced airway inflammation as well as inhibited Th2 cytokine expression including IL-5 and IL-13 [254].

## 6. Human Parainfluenza Virus (hPIV)

### 6.1. hPIV Virology and Disease

Within the order Mononegavirales, the Paramyxoviridae family is a large group of viruses that cause significant human and veterinary disease in addition to the Pneumoviridae family [255]. Being members of the family Paramyxoviridae, hPIVs, which are characterized by an enveloped virion and single-stranded negative-sense RNA genome, were first isolated in 1950’s in patients with lower respiratory tract disease [256]. hPIVs are genetically and antigenically divided into types 1 to 4 along with further described subgroups or subtypes [257,258,259]. There are two genera of hPIV, Respirovirus (hPIV-1 and hPIV-3) and Rubulavirus (hPIV-2 and hPIV-4). Mouse Sendai virus (SeV), which is responsible for a highly transmissible respiratory tract infection, is a member of genus Respirovirus.

hPIVs cause both upper and lower respiratory infections with clinical presentations including cough, acute laryngotracheobronchitis (croup), bronchiolitis, tracheobronchitis, pneumonia and, rarely, neurologic disease [260,261,262,263]. hPIV-induced respiratory infection contributes to hospitalization in children under age 5 [264,265]. hPIV 1 to 3 are commonly detected, while infants younger than 6 months are particularly vulnerable to hPIV-3 infection [266]. Cases of hPIV4 infection are rare. hPIVs have been detected in asthma patients [267,268,269] but there are few studies correlating hPIV infection and asthma development or exacerbation.

### 6.2. Animal Models of hPIV Infection

hPIVs infect many different animals both naturally and under experimental conditions. In hamsters and cotton rats, hPIV3 replication peaks within 48 h, with histologic changes including epithelial damage and inflammatory infiltrates [24,26]. Interestingly, histologic changes vary in different cotton rat strains following hPIV infection: Sigmodon hispidus develops bronchiolitis, while Sigmodon fulviventer develops interstitial pneumonia [24]. After experimental infection, hPIV viral antigen appears in bronchial and bronchiolar epithelial cells, macrophages and alveolar type II pneumocytes [24]. hPIV3 also replicates in the larynx and causes laryngotracheitis in the cotton rat [270]. hPIV3 causes fatal disease in newborn ferrets [27,271].

The hPIVs poorly infect mice [272]. Instead, experimental infection with mouse parainfluenza type 1 Sendai Virus (SeV) allows for high viral replication and a pattern of infection and illness that resembles hPIV infection [28,70,71]. The primary infection of SeV in the living mice has been determined using a recombinant SeV expressing luciferase reporter, [273]. Contact transmission of SeV leads to robust viral titers in the upper respiratory tract with later spread to the lungs [273]. The primary infection after airborne transmission starts either in the nasopharynx or in the trachea [273]. SeV antigens are detected in both bronchiolar and alveolar epithelial cells in infected rats and mice [28,74]. SeV increases epithelial-derived IL-12 p40 production which is associated with increased mobility and mortality [70]. SeV also targets the macrophage and induces apoptosis during its replication [71]. SeV infection causes dose-dependent effects in mice ranging from no effect to reversible bronchiolitis to lethal bronchopneumonia. Inflammatory infiltrates include neutrophils, lymphocytes and monocytes/macrophages [28,70,274]. The intercellular adhesion molecule 1 (ICAM-1) has been associated with immune cell recruitment during SeV infection and ICAM-1 deficient mice are protected against acute SeV infection [28,275]. In addition, the CCL5-CCR5 axis is essential to maintain the viral-clearance function of macrophages and to drive migration of lung conventional DCs to the lymph nodes [71,276].

### 6.3. SeV Model of Asthma Development

After SeV acute infection of naïve mice, there is a delayed but permanent switch to chronic airway responses which are characterized by airway hyperresponsiveness and chronic goblet cell hyperplasia which develop by 7 weeks of age [28,277]. In these mice, expression of IL-13 and Muc5ac is observed weeks after initial SeV infection. The major cellular source of IL-13 changes from the CD4+ T cell at day 21 after infection to macrophages at 7 weeks after infection [277]. NKT cells and macrophages are required for maximal IL-13 production [277]. Moreover, SeV infection increases type I IFN receptor-dependent expression of FcεRI and production of CCL28 on/by mouse lung dendritic cells, leading to recruitment of IL-13-producing Th2 cells [278]. Recent studies also suggests the importance of CD49d+/cysteinyl leukotriene receptor 1 (CysLTR1) + polymorphonuclear neutrophils for FcεRI expression on mouse lung DCs and subsequent chronic asthma development [279,280,281]. In addition, the persistent infection of SeV in the nerve tissue has been linked to chronic asthma development [282]. In guinea pigs, SeV infection alters vagal afferent innervation in the airways, with increased tachykinin expression in both nociceptive-like and non-nociceptive neurons [30]. SeV may also cause dysfunction of inhibitory M2 muscarinic receptors on the airway parasympathetic nerves [283].

The effects of early life SeV infection on later asthma development has also been extensively studied in rats. SeV infection induced necrotizing bronchiolitis and interstitial pneumonia as well as transient airway obstruction and hyperresponsiveness in five day-old, 25 day-old and adult rats [74,76,284]. SeV infection in young rats also leads to persistent lung morphologic changes including alveolar dysplasia, bronchiolar hypoplasia and bronchiolar mural fibrosis [29,75,77,284,285,286]. SeV infection of five and 25 day-old rats causes airway hyperresponsiveness for up to 10 weeks [29,77,285]. The induced asthma-like phenotypes in young rats are strain-dependent [29]. Compared to SeV-infected Brown Norway rats which show increased thickness of bronchiolar wall, persistent pulmonary dysfunction and airway hyperresponsiveness, the Fischer 344 rats are more resistant to virus-induced alternations in pulmonary function abnormalities [29]. In addition, the infected Brown-Norway strain but not the Fischer strain showed a persistent level of IL-13 after early-life SeV infection [287]. Persistent viral infection has been noticed in both infected rat strains and there is no significant difference of viral replication [287]. Young Brown-Norway rats respond differently than Fischer rats to acute SeV infection, as indicated by lower IFN-γ levels in bronchoalveolar lavage fluid, increased expression of TGF-β and differential activation of NFκB signaling [29,288,289,290,291].

## 7. Human Coronavirus (HCoV) and Human Bocaviruses (HBoV)

Members of the Coronaviridae family, OC43, 229E, NL63 and HKU1, have been associated with self-limiting respiratory tract infections in human. On the other hand, severe acute respiratory syndrome (SARS)-CoV and Middle East respiratory syndrome (MERS)-CoV, cause severe respiratory disease in human. Most animal models have been established for the study of SARS-CoV and MERS-CoV which are less relevant to asthma.

HBoV was first isolated in 2005 and has been detected in both the respiratory tract and gastrointestinal tract [292,293,294]. HBoVs belong to the family Parvoviridae, which consists of a group of small non-enveloped single-stranded DNA viruses. HBoV has been found alone in patients with respiratory complaints but more often, it is found in combination with other common respiratory viruses such as HRV and RSV [295]. Though the presence of HBoV has been associated with clinical manifestations including rhinorrhea, pneumonia, bronchiolitis, acute wheezing and asthma exacerbation [293,295,296,297], HBoV pathogenicity remains to be fully clarified mainly due to the lack of animal models. The first trial of HBoV infection in ferret lung has recently been performed [298].

## 8. Closing Remarks

Respiratory viral infections are strongly associated with asthma exacerbations. Further, wheezing associated respiratory viral infections in early life have been linked to asthma development. Investigators have therefore studied human respiratory viral infections in small animals, with the mouse being the predominant species studied.

In this paper we have touched on the difficulties and limitations of animal models for determining pathways of virus-induced asthma. To review and expand on this point, small animals are less permissive to human viruses and usually require a high dose of inoculum for viral respiratory infection, in particular viral replication. The barriers to replication may include differences in viral receptors, mucosal barriers, signaling molecules and other mechanisms. As a result, the kinetics of viral replication and clearance in small animals are often different from natural human infection. In addition, human viral infections in small animals are mostly asymptomatic, which makes clinical correlation difficult. What then, can we learn from small animal models of viral infection?

Certainly, studies of human viral replication and clearance in small animals must be regarded with some hesitation. On the other hand, the inflammatory response driving respiratory viral diseases appears intact in small animals. In contrast to airway epithelial cells which support viral replication, recent data from our lab [47,55,56,124,126,127,134] and others [129,277,299,300,301] suggest that the inflammatory response to respiratory viral infection in large part arises from and depends on innate immune effector cells, rather than epithelial cells (Figure 2). These include exudative macrophages, innate lymphoid cells, NKT cells and plasmacytoid dendritic cells. 

Thus, despite limitations, studies have yielded valuable information about viral pathogenesis and potential mechanisms of asthma exacerbation and development. Recent studies have identified specific roles for epithelial-derived innate cytokines and their cellular targets. In some cases, cell and molecular mediators identified in animal models have been confirmed in human subjects. Further experimentation in mouse models will undoubtedly contribute to the development of new therapeutic interventions for asthma.

## Figures and Tables

**Table 1:** Asthma-associated respiratory viruses and infection susceptibility in small animals.

Taxonomy				Species				
Order	Family	Subfamily and Genus	Species	Mice	Rats	Guinea Pigs	Hamsters	Ferrets
*Picornavirales*	*Picornaviridae*	*Enterovirus*	*Rhinovirus A-C*	HRVA-1B [4,5] HRV-A16 [4]	HRVA-16 [6] HRVB-14 [7]			
			*Enterovirus D*	EV-D68 [8,9,10] Fermon and epidemic strains	EV-D68 [11] Fermon and epidemic strains			EV-D68 [12] Fermon strain
*Mononegavirales*	*Pneumoviridae*	*Orthopneumovirus*	*Human orthopneumovirus*	human RSV-A [13]	human RSV-A [14]	human RSV-A [15]	human RSV-A [16]	human RSV-A [17]
			*Murine orthopneumovirus*	PVM [18]				
		*Metapneumovirus*		hMPV [19]	hMPV [20]	hMPV [21]	hMPV [21,22]	hMPV [23]
	*Paramyxoviridae*	*Respirovirus*	*Human Respirovirus* 1,3		hPIV3 [24]	hPIV3 [25]	hPIV3 [26]	hPIV3 [27]
			*Murine Respirovirus*	SeV [28]	SeV [29]	SeV [30]	SeV [31]	
		*Rubulavirus*	*Human Respirovirus 2,4*					

**Table 2:** Mouse models for respiratory viral infection.

Viruses	Species ^a^	Age	Gender ^b^	Route ^c^	Virus Strains/Isolates	Inoculum ^d^	Dura-Tion ^e^	Detection Method	Applications and Major Observations	Reference
HRV	BALB/c mice	6 weeks	F	i.n.	HRVA-1B	5 × 10^6^ TCID_50_	72–96 h	Viral titer/Viral RNA/ v(-) RNA	HRV induced airway inflammation with inflammatory infiltrates and increased expression of cytokines, chemokines and IFNs as well as mucus-related proteins	[4]
	huICAM BALB/c mice	N/A	N/A	i.n.	HRVA-16	5 × 10^6^ TCID_50_	N/A	Viral RNA/		
	C57BL/6 mice	6–8 weeks	F	i.n.	HRVA-1B	5 × 10^6^ TCID_50_	96 h	Viral RNA/ v(-) RNA	HRV induced phosphatidylinositol 3-kinase dependent airway inflammation and airway responsiveness	[5]
	BALB/c mice	6 days	N/A	i.n.	HRVA-1B	2 × 10^6^ TCID_50_	7 d *	Viral RNA/	Neonatal model of HRV infection. Early-life HRV infection induced the development of asthma like phenotype which is IL-13 dependent and ILC2 expansion	[53,56]
EV-D68	BALB/c mice	8–12 weeks	F	i.n.	US/MO/14-18947	5 × 10^6^ epfu	96 h	Viral RNA	EV-D68 infection induces IL-17-dependent airway inflammation and hyperresponsiveness which is greater than HRV in naïve mice	[9]
Human RSV	20 strains	3 days	N/A	i.n.	Long strain	10^3.3^–10^3.7^ pfu	N/A	Viral titer	The susceptibility of HRSV infection is different among 20 strains of inbred 3-day old mice. DBA/2N is the most permissive strain.	[13]
	8 strains	8–10 weeks	F	i.t.	A2 strain	4 × 10^7^ pfu	N/A	Viral titer	AKR/J is the most permissive among eight strains of adult mice for human RSV infection, C57BL/6 is the most resistant	[57]
	BALB/c mice	1 day-32 weeks	N/A	i.n.	A2 strain Long strain	10^4^–10^7^ pfu	8–12 d *	Viral titer	Human RSV causes weight loss, bronchiolitis, pneumonia and increased CD8+ T cell and NK cell responses. RSV reinfection after early primary infection causes more severe disease. RSV persists in the lung.	[58,59,60,61,62,63,64]
	BALB/c mice	6–8 weeks	N/A	i.n.	6 clinical isolates	10^5^ pfu	N/A	N/A	The clinical RSV isolate A2001/2-20 caused more severe lung dysfunction, airway responsiveness, IL-13 and mucus expression	[65]
	BALB/c mice	8 weeks	N/A	i.n.	A2001/2-20 strain	3 × 10^6^ pfu	N/A	N/A	In adult mice, RSV A2001/2-20 strain triggers expansion of IL-13 expressing ILC2s which is TSLP dependent.	[66]
	BALB/c mice	5 days	N/A	i.n.	A2 strain rA2-19F strain	10^4.68^ TCID_50_/g	6 d *	Viral RNA Viral titer	Neonatal infection with recombinant human RSV rA2-19F causes increased Th2 responses at primary infection and augmented airway hyperreactivity, mucus hyperproduction and eosinophilia during reinfection compared to the A2 strain.	[54]
HMPV	BALB/c mice	4–8 weeks	N/A	i.n.	NL/100 strain C-85473 strain CAN98-75 strain CAN98-83 strain	5 × 10^5^–10^8^ pfu	21–60 d	Virus titer	hMPV replicates with a biphasic growth kinetic and persists in the lung. hMPV induces both Th1 and Th2 responses. hMPV infection causes clinical symptoms (dyspnea and weight loss) as well as mucus production, airway hyperresponsiveness and obstruction.	[19,22,67,68,69]
	9 strains	5–6 weeks	N/A	i.n.	TN/96-12 strain	10^5^ pfu	N/A	Virus titer	DBA/2 mice is the most permissive strain for hMPV.	[21]
HPIV/ SeV	C57BL/6 mice	6–12 weeks	N/A	i.n.	Fushimi strain	2 × 10^5^ pfu/ 5000 EID_50_	N/A	N/A	Acute SeV infection leads to chronic airway hyperresponsiveness and mucus metaplasia, along with increased and maintained IL-13 expression by CD4+ T cells and macrophages.	[28,70,71]
	BALB/c mice	6–8 weeks	F	i.n.	N/A	500 EIU	N/A	N/A		[72]

^a^ Different inbred mice strains have been used to compare their susceptibility for the infection of a certain respiratory virus strain. ^b^ For gender, F = female, M = male, N/A = not applicable. ^c^ Animals were inoculated by the intranasal (i.n.), intratracheal (i.t.) routes. ^d^ Viral titers were quantified by different units: plaque forming units (pfu), 50% tissue culture infectious dose (TCID_50_), 50% egg infectious dose (EID_50_), egg infectious unit (EIU). ^e^ The symbol “*” indicates the duration of the infection ended due to the termination of time course study. N/A = not applicable.

**Table 3:** Other small animal models of respiratory viral infection.

Viruses	Species	Age	Gender	Routes	Virus Strain/isolates	Inoculum	Duration	Detection Method	Reference
HRV	Cotton rat *(Sigmodon hispidus)*	8 weeks	F/M	i.n.	HRVA-16	10^7^ pfu	48 h	Viral titer/v(-) RNA	[6]
	Cotton rat *(Sigmodon hispidus)*	4–6 weeks	N/A	i.n.	HRVB-14	3.97 × 10^6^ pfu	48 h	Viral titer/v(-) RNA	[7]
EV-D68	Cotton Rat *(Sigmodon hispidus)*	6–8 weeks	F	i.n.	Fermon	10^6^ pfu	N/A	Viral titer	[11]
					VANBT/1	10^6^ pfu	24 h	Viral titer/v(-) RNA	
					US/MO/14/18949	10^6^ pfu	N/A	Viral titer	
	Ferret *(Mustela putorius furo)*	N/A	M	aerosol spray	Fermon	10^4.5^ CCID50	15 d *	Viral RNA	[12]
Human RSV	Cotton rat *(Sigmodon hispidus)*	1–28 days	N/A	i.n.	Long	10^4^ pfu	7 d	Virus titer	[14]
	Ferret *(Mustela putorius furo)*	1–28 days	N/A	i.n.	Long	3.6 × 10^3^ pfu	9 d	Virus titer	[17]
	guinea pigs (Cam Hartley)	N/A	Female	i.n.	Long	3.9 × 10^3^ pfu	14 d *	Viral titer	[15,73]
hMPV	Cotton rat *(Sigmodon hispidus)*	5 weeks	N/A	i.n.	NL/100	10^6^ pfu	N/A	N/A	[22]
		5–6 weeks	N/A	i.n.	TN/96-12	10^5^ pfu	8 d	Virus titer	[21]
		N/A	N/A	i.n.	26583(subtype A) 26575(subtype B)	9 × 10^5^ TCID_50_	14 d	N/A	[20]
	Ferrets (Mustela putorius)	5 weeks	N/A	i.n.	NL/100	10^6^ pfu	N/A	N/A	[22]
	Hamster (Mesocricetus auratus)	5 weeks	N/A	i.n.	NL/100	10^6^ pfu	N/A	N/A	[22]
		5–6 weeks	N/A	i.n.	TN/96-12	10^5^ pfu	N/A	Virus titer	[21]
	guinea pigs (*Cavia porcellus*)	5–6 weeks	N/A	i.n.	TN/96-12	10^5^ pfu	N/A	Virus titer	[21]
hPIV	Cotton rat *(Sigmodon hispidus)*	N/A	N/A	i.n.	hPIV3/F518	10^5.8^ pfu	8 d	Virus titer	[24]
	Cotton rat *(Sigmodon fulviventer)*	N/A	N/A	i.n.	hPIV3/F518	10^5.8^ pfu	8 d	Virus titer	[24]
	Ferret	1 day	N/A	Aerosolization	HPIV3/224466 HPIV3/C243	N/A	N/A	N/A	[27]
	Hamster *(Mesocricetus auratus)*	N/A	N/A	i.n.	hPIV3 strain C243	100–6000 pfu	7–8 d	Virus titer	[26]
SeV	Crl:CD(SD) rat	5–25 days	N/A	i.n.	N/A	10^2.4^ TCID_50_	7–10 d	Virus titer	[74]
	Crl:CD(SD) rat	5–25 days	N/A	Aerosol exposure	N/A	1.34 pfu/mL gas	N/A	N/A	[75]
	Crl:CD(SD) rat	10 weeks	Male	Aerosol exposure	SeV P3193	1–3 pfu/mL gas	N/A	N/A	[76]
	Crl:CD(SD) rat	5 days	N/A	Aerosol exposure	SeV P3193	1–2 pfu/mL gas	N/A	N/A	[77]
	Brown Norway rat	5–25 days	N/A	aerosol exposure	SeV P3193	1–3 pfu/mL gas	N/A	N/A	[29]
	Fischer 334 rat	5–25 days	N/A	aerosol exposure	SeV P3193	1–3 pfu/mL gas	N/A	N/A	
	Guinea pigs	N/A	Male	i.n.	SeV52	5 × 10^5^ TCID_50_	N/A	N/A	[30]

The symbol “*” indicates the duration of the infection ended due to the termination of time course study. N/A = not applicable.
